# Dangers of the Use of Ergot in Progressive Locomotor Ataxy

**Published:** 1883-09

**Authors:** 


### Dangers of the Use of Ergot in Progressive Locomotor Ataxy.

Grasset reports a case of this affection, aged 38, widower, without children, father suffered from rheumatism; had committed venereal excess; no history of syphilis. Four years ago rheumatoid pains, and headaches lasting for two years; then, sudden paralysis of the third nerve on the right side, with diplopia and vertigo. Two or three months later disturbance of motility, heaviness in walking and incoördination; light darting pains in the limbs, but very severe in the rectum and perineum.

In 1880 the disease was well marked (disturbance of sensibility of the lower extremities), atony of the sphincters, incoördination, etc. First season at St. Malo. In 1881, second season; decided improvement. The sphincters act almost normally; the patient walks well; he spends the winter at Hyéres, always better. At the end of the winter he becomes more easily tired, more heavy; however, this condition improves, and he goes to Paris to consult Charcot, who had already seen him. He is ordered at first 25 centigrammes of ergot a day; increasing steadily at the rate of 5 centigrammes a day, until 1 gramme is taken. The patient was ordered to continue at the rate of 1 gramme a day for three days, then to stop and take in place of it the nitrate of silver. The patient returns to Marseilles, and continues the treatment without consulting any local physician. He takes the ergot, and the second of the days when he is to take one gramme daily, he has paralysis of the four extremities and of the voice; he cannot stir, and says he felt the paralysis coming on for a day or two past. The sensibility is much diminished in the paralyzed limbs, even in the upper extremities, which had up to that time always been intact. He has no pain anywhere, but cannot move; he can neither rise nor remain seated, but is condemned to absolute immobility. He stops the ergot immediately; a steady improvement at once begins. At the time of the examination by Grasset the arms have almost completely recovered, the patient writes. The voice is entirely restored. He can seat himself very easily on his bed, and remains seated. Some urinary difficulty persists, no disturbance of the rectum, has incoördination in the movements of the lower lids, especially when the eyes are closed; slight ptosis of the upper lid; no patellar tendon reflex; very slight disturbance of the sensibility of the lower extremities.

The author believes himself justified in attributing this sudden and marked change for the worse to the noxious influence of ergot. The patient at the time he began taking it had been steadily improving; during its continuance paralysis comes on; the remedy is stopped, and, at once, there is an improvement. Nor has the dose given been at all excessive; the majority of authors, who recommend it, give more. Hammond advises from five to six grammes daily continued for several months; Erb, who scarcely recommends it, cites Waldmann and says he gives from one to two grammes daily. Since the labors of Brown-Sequard, ergot has been considered as indicated by hyperæmia of the spinal cord, and the fact that the patient was experiencing some fatigue, and that therefore an increase of the trouble was imminent, would seem to have fully justified its use. Yet the result was disastrous.

The author then quotes from Nothnagel and Rossbach with reference to the physiological action of ergot on the spinal cord: "In warm-blooded animals, the aqueous extract of ergot in comparatively small doses, and doubtless sclerotic acid also, produce anæsthesia, and disturbances of coordination; in large doses they cause paralysis, during which the animal manifests neither voluntary nor reflex movements and is insensible to the most painful irritation."

But the last results published by Tuczek are of particular value with regard to the case of the author. Following a severe epidemic of spasmodic ergotism, which affected 500 persons in a country of 2,500, 29 persons affected with psychical troubles entered the hospital at Marburg. Tuczek's report embraces the last eighteen of these, on four of which an autopsy was made.

All the patients manifested symptoms of lesion of the posterior columns of the cord; in the four cases which succumbed, this lesion was proven anatomically. In all cases, the knee reflex was absent and did not reappear when the cure in other respects seemed complete. The other spinal symptoms were: Paræsthesias, formication, darting pains, anæsthesia, analgesia, symptom of Romberg ataxia. In some cases the complete picture of dorsal tabes developed. The four spinal cords examined belonged to individuals of 9, 16, 20 and 33 years of age. The lesion of the posterior columns occupied the entire length of the cord; in two cases it could be followed into the medulla; it was symmetrical on the two sides and was limited to Burdach's columns. In the most marked case, where the lesion involved nearly the whole of the posterior columns, it was seen that the prócess was older and more marked in the lateral fasciculi. Tuczek concludes that ergot had produced in all the individuals a systemic spinal lesion, absolutely identical with tabes dorsalis. A great number of experiments in animals had failed, but he had seen the hypodermic injection of sclerotic acid produce a veritable ataxy in rabbits.

This last fact, if confirmed, is of great value to experimental pathology. It is most important that ergot develops in certain cases posterior spinal lesions comparable to those of locomotor ataxy as well as in their pathology as their symptoms. For this reason the author urges the most stringent precautions in the treatment of locomotor ataxy by ergot. J. E.

—*From the Progres Medical, in the Cincinnati Lancet and Clinic.*

